# ‘Not finding causal effect’ is not ‘finding no causal effect’ of school closure on COVID-19

**DOI:** 10.12688/f1000research.111915.2

**Published:** 2024-04-16

**Authors:** Akira Endo

**Affiliations:** 1 The Centre for Mathematical Modelling of Infectious Diseases, London School of Hygiene & Tropical Medicine, London, WC1E 7HT, UK; 2 Department of Infectious Disease Epidemiology, London School of Hygiene & Tropical Medicine, London, WC1E 7HT, UK; 3 School of Tropical Medicine and Global Health, Nagasaki University, Nagasaki, 852-8521, Japan; 4 Saw Swee Hock School of Public Health, National University of Singapore, Singapore, 117549, Singapore

**Keywords:** COVID-19, school closure, Japan, causal inference, reanalysis

## Abstract

In a paper recently published in *Nature Medicine*, Fukumoto et al. tried to assess the government-led school closure policy during the early phase of the COVID-19 pandemic in Japan. They compared the reported incidence rates between municipalities that had and had not implemented school closure in selected periods from March–May 2020, where they matched for various potential confounders, and claimed that there was no causal effect on the incidence rates of COVID-19. However, the effective sample size (ESS) of their dataset had been substantially reduced in the process of matching due to imbalanced covariates between the treatment (i.e. with closure) and control (without closure) municipalities, which led to the wide uncertainty in the estimates. Despite the study title starting with “No causal effect of school closures”, their results are insufficient to exclude the possibility of a strong mitigating effect of school closure on incidence of COVID-19. In this replication/reanalysis study, we showed that the confidence intervals of the effect estimates from Fukumoto et al. included a 100% relative reduction in COVID-19 incidence. Simulations of a hypothetical 50% or 80% mitigating effect hardly yielded statistical significance with the same study design and sample size. We also showed that matching of variables that had large influence on propensity scores (e.g. prefecture dummy variables) may have been incomplete.

## Introduction

A paper recently published in *Nature Medicine*, Fukumoto *et al.*, tried to assess the government-led school closure policy during the early phase of the COVID-19 pandemic in Japan. They compared the reported incidence rates between municipalities that had and had not implemented school closure in selected periods from March–May 2020, where they matched for various potential confounders, and claimed that they found no causal effect on the incidence rates of COVID-19. School closure as a means to control outbreaks has been studied mostly for influenza prior to the emergence of COVID-19, which generally suggested low-to-moderate effects, but the evidence on other respiratory infections including coronavirus diseases has been limited ( Viner *et al.*, 2020). Sometimes decisions need to be made in the lack of sufficient evidence in the earliest phase of the pandemic; nonetheless, such decisions should undergo retrospective policy assessment to provide insights and refinement for future pandemic responses.

One of the challenges in this type of analysis of the early COVID-19 epidemic in Japan is the limited statistical power due to low case counts. During the first wave of the epidemic from February to June 2020 that overlapped with the study period of Fukumoto *et al.*, Japan never observed more than 1,000 COVID-19 cases per day. As a result, out of the total 79,989 municipality-level daily counts from the 847 municipalities included, 99.9% were less than 10 cases per day (Figure S2 of the original study). Moreover, the matching technique used to minimise confounding has a known side effect of limiting statistical power, especially when there is little overlap in the covariates between arms ( King *et al.*, 2017).

Unfortunately, the analysis in Fukumoto *et al.* appears to suffer from these issues. The study title says “No causal effect”, which is a rather strong statement given the substantial uncertainty in their estimates. As the saying goes, “absence of evidence is not evidence of absence”—when the uncertainty range covers practically meaningful values, it should not be prematurely concluded that there is “no effect” just because the effect estimates are statistically insignificant. Here I highlight limitations of the analysis and discuss possible factors that may have rendered the study underpowered.

## Relative ATC and ATT estimates

The original study measures the effect of school closures as the absolute difference in incidence rates between the treatment and control municipalities. However, the theoretical ground is unclear for assuming a fixed additive effect of school closures on the incidence rate per capita. Infectious disease risks are inherently dynamic; more current infections in a population would result in a greater risk of infection among susceptible individuals through increased encounters with infectious others. This means that the effect of school closures, which intended to reduce contacts at schools, should also depend on the baseline incidence in the population because the risk of infection averted would be the reduction in contacts multiplied by the probability that the contacts were otherwise with infectious individuals. The effect estimates relative to the baseline incidence would therefore be a more relevant and interpretable measure for assessment of its practical use. It should also be noted that since incidence rates can only take non-negative values, the absolute mitigating effect of school closure can only be as high as the average incidence rate in the control group.

**Figure 1.**
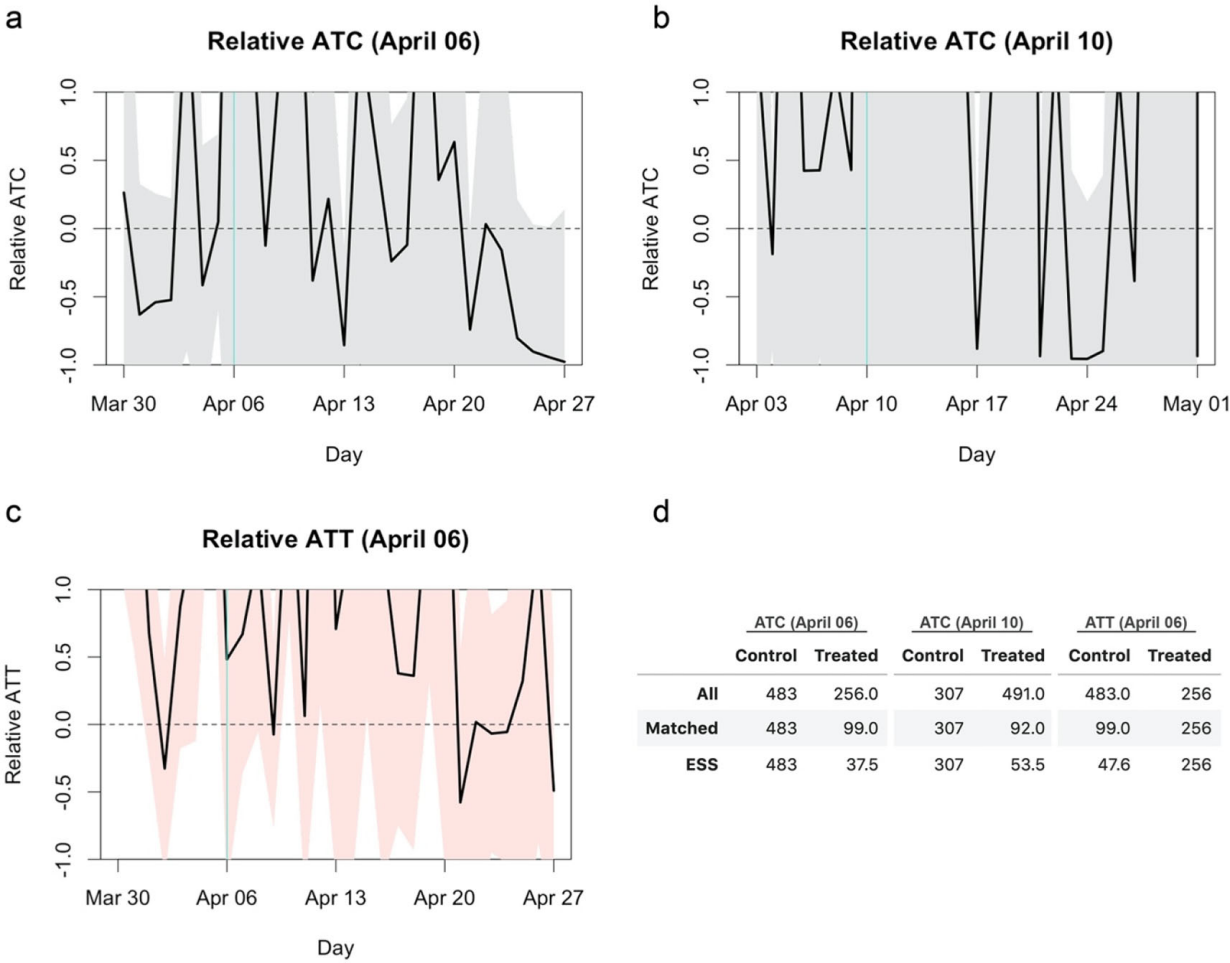
Relative average treatment effect on the control (ATC) and average treatment effect on the treatment (ATT). The turquoise vertical lines represent the date of treatment (school closure). The black lines and shaded areas represent the mean effect and 95% confidence intervals, respectively. (a) Relative ATC for the closure as of April 6, 2020. (b) Relative ATC for the closure as of April 10, 2020. (d) Relative ATT for the closure as of April 6, 2020. (d) Comparison of sample sizes. The number of all samples included for matching, the number of unique samples matched to at least one other sample and the effective sample size (ESS) of the matched samples are shown.

I rescaled the reported average treatment effects (average treatment effect on the control: ATC; and average treatment effect on the treatment: ATT) and their confidence intervals relative to the average outcome (incidence rate per capita) in the control group ( Figure 1). The confidence intervals of the relative ATC and ATT cover most of the regions from 100% reduction to 100% elevation, suggesting the underpowered nature of the original study. An effect of 50% reduction (i.e. -50% relative effect), which most experts would agree is of practical significance, or even complete reduction (i.e. -100%) was within the confidence intervals over the substantial part of the period of interest. The effective sample size (ESS; a proxy measure for the amount of information contained in weighted samples ( Shook-Sa and Hudgens)) of the matched arms of around 40–50 ( Figure 1d) was likely insufficient to find a statistical significance because incidence of infectious diseases typically exhibits higher dispersion than independent- and identically-distributed settings due to its self-exciting nature (i.e. an increase in cases induces a further increase via transmission).

## Statistical power demonstration with assumed causal mitigating effect of 50%/80%

**Figure 2.**
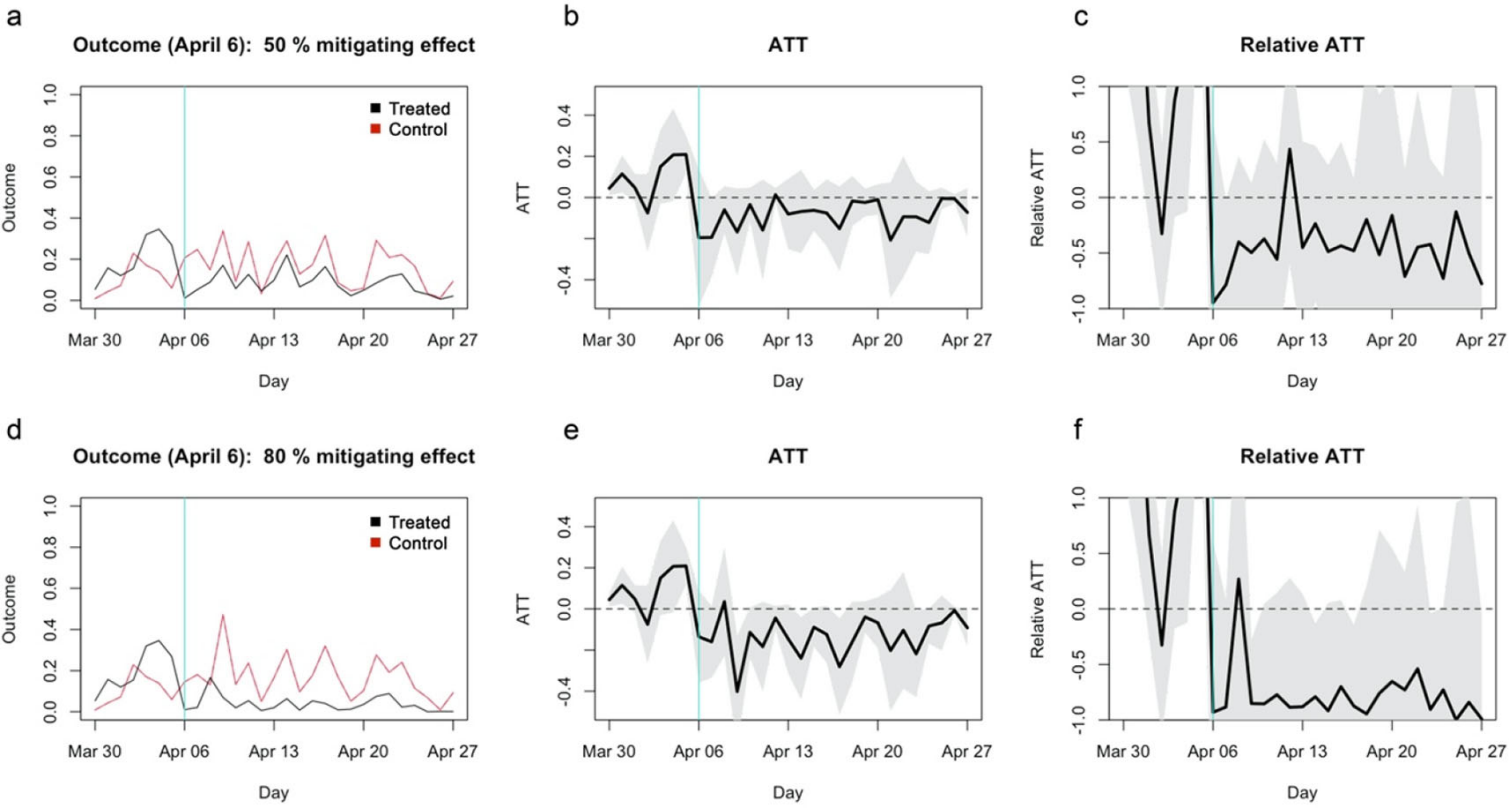
Simulated average treatment effect on the treatment (ATT) estimates assuming 50%/80% mitigating effects. (a) The average outcome (incidence per capita) of the matched treatment (black) and control (red) groups for closure as of April 6, 2020. (b) Absolute ATT estimates (black line) and 95% confidence intervals (shaded area) for closure as of April 6. (c) Relative ATT estimates and 95% confidence intervals for closure as of April 6. (d)–(f) Those for closure as of April 10.

**Figure 3.**
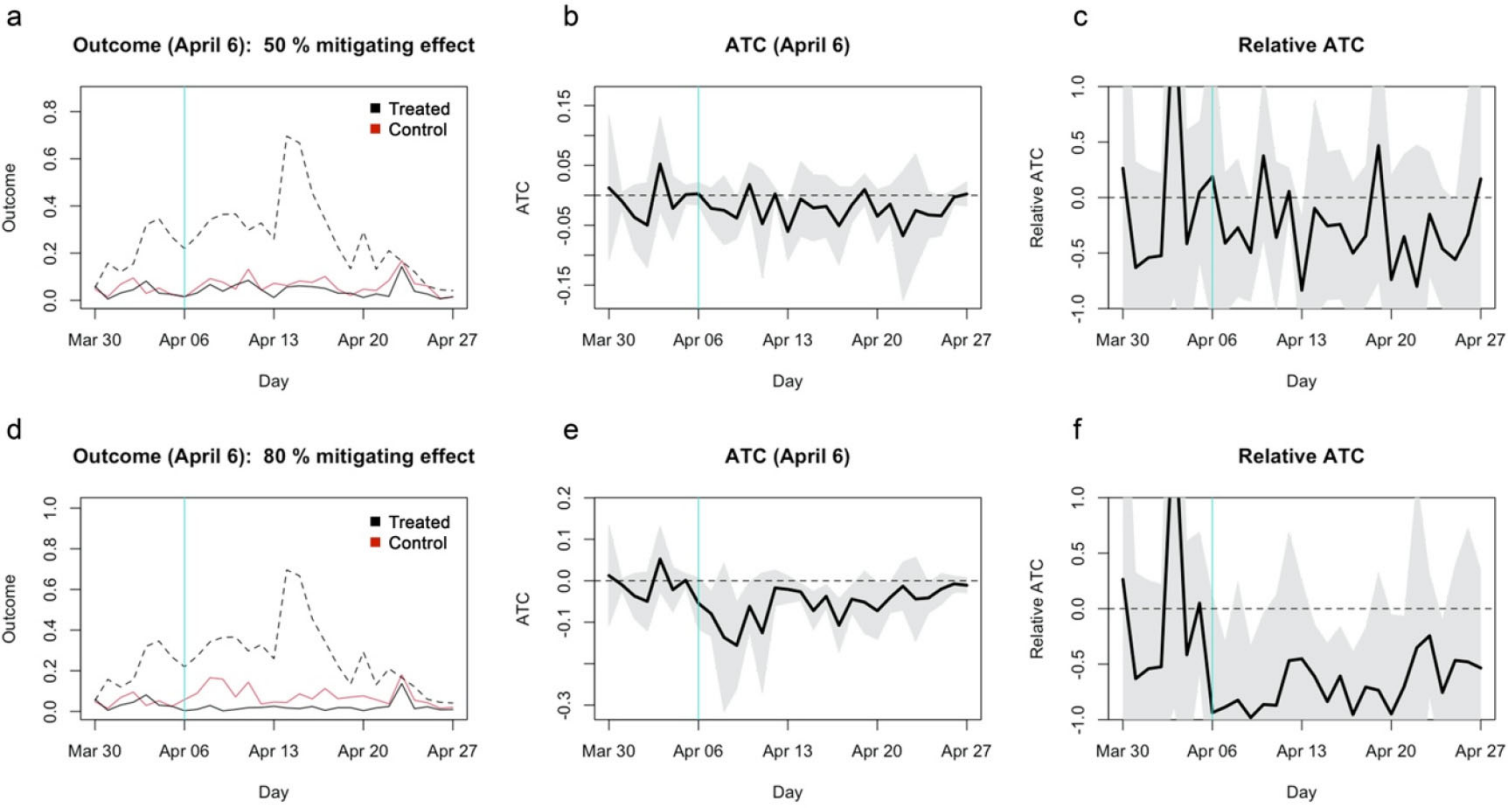
Simulated average treatment effect on the control (ATC) estimates assuming 50%/80% mitigating effects. (a) The average outcome (incidence per capita) of the unmatched treatment (dashed), matched treatment (black) and control (red) groups for closure as of April 6, 2020. (b) Absolute ATC estimates (black line) and 95% confidence intervals (shaded area) for closure as of April 6. (c) Relative ATC estimates and 95% confidence intervals for closure as of April 6. (d)–(f) Those for closure as of April 10.

To further examine the statistical power of the study, I artificially modified the dataset such that school closure has a 50% or 80% mitigating effect on the incidence rate per capita. On the treatment reference date (April 6) and onward, the expected incidence rate of each municipality in the treatment group was assumed to be 50%/20% that of the matched control municipality plus Poisson noise (see Extended data: Supplementary document for details). The results suggested that, even with as much as 50%/80% mitigating effect, the approach in the original study might not have reached statistical significance ( Figure 2). The absolute ATT for the 50% mitigating effect ( Figure 2b) appears similar to what were referred to as “no effect” in the original study. ATT for the 80% mitigating effect was also statistically insignificant ( Figure 2c and 2d), suggesting that the study was underpowered to find even moderate to high mitigating effects, if any. ATC estimates also yielded similarly insignificant/barely significant patterns ( Figure 3).

## Separation of propensity scores

**Figure 4.**
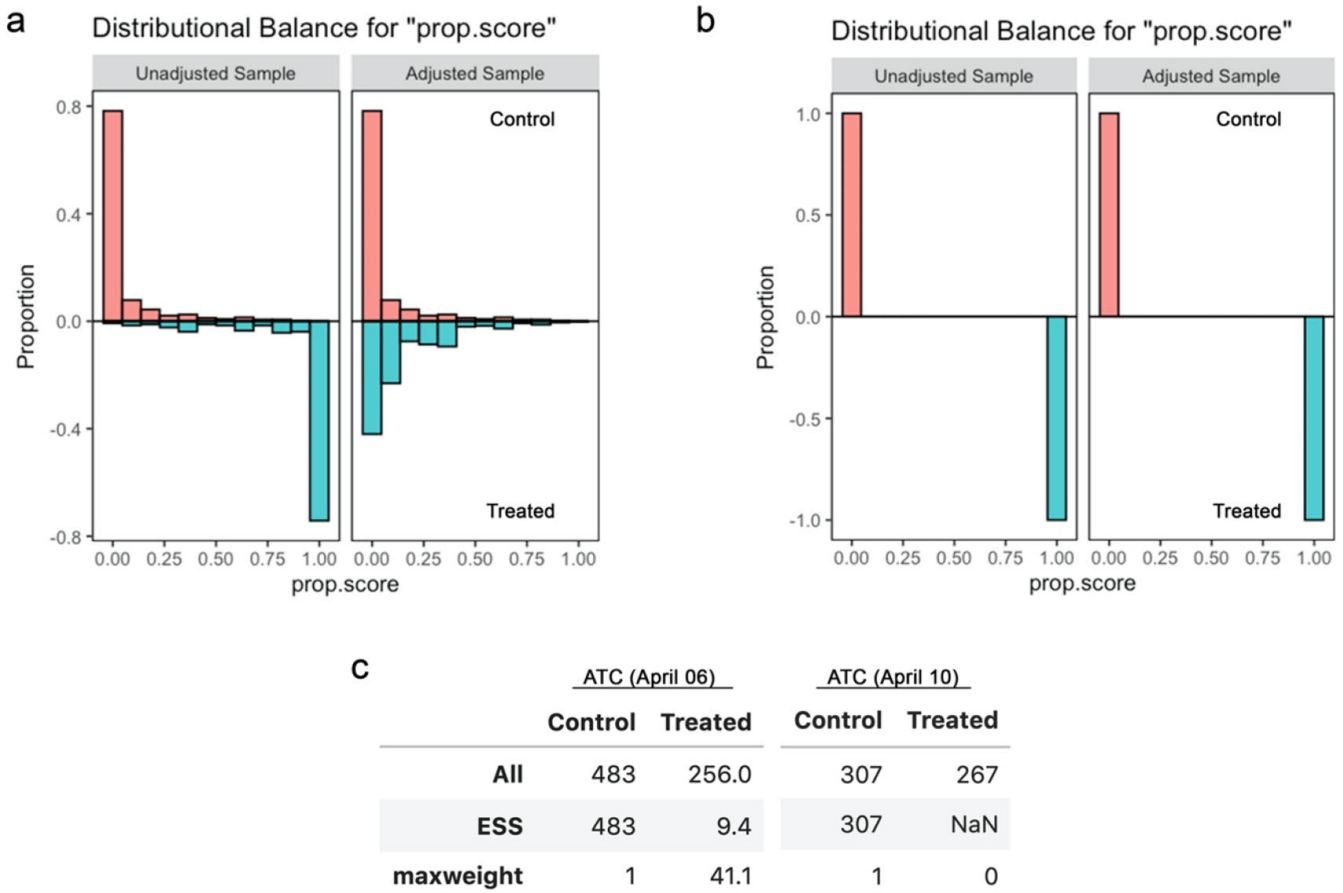
Propensity scores and effective sample sizes for the inverse probability weighting analysis in the original study. (a) Balance of propensity scores before and after matching for school closure as of April 6, 2021. (b) Balance of propensity scores before and after matching for school closure as of April 10, 2021. (c) All and effective sample sizes and the maximum weight among the samples. The effective sample size of NaN indicates that the all samples received zero weights.

**Figure 5.**
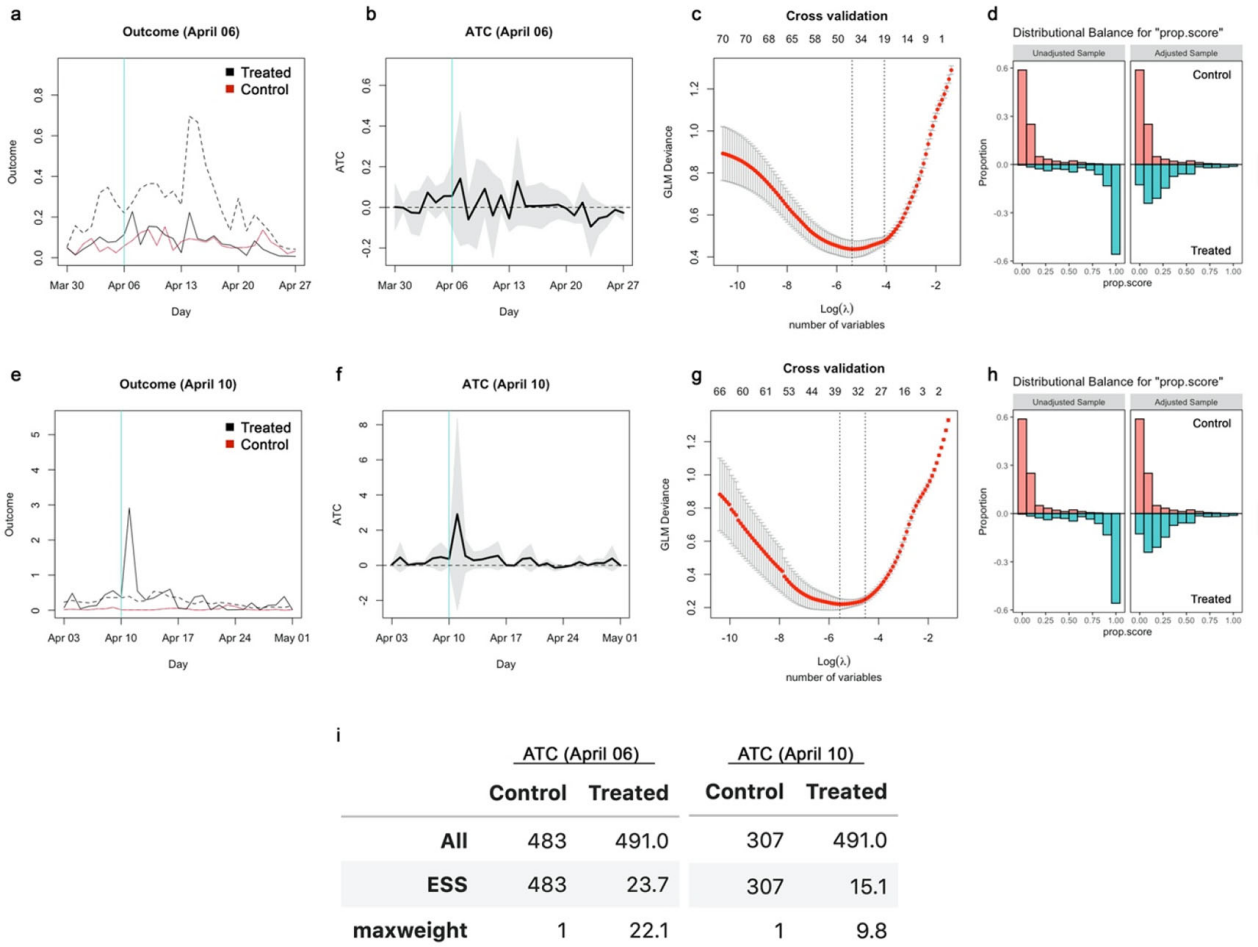
Inverse probability weighting with Lasso regularisation. (a) The average outcome (incidence per capita) of the unmatched treatment (dashed), matched treatment (black) and control (red) groups for closure as of April 6, 2020. (b) Absolute ATC estimates (black line) and 95% confidence intervals (shaded area) for closure as of April 6. (c) Result of 10-fold cross validation. The x-axis represents the logarithm of the regularisation coefficient *λ* for each model; the number of included variables is also displayed above the panel. The left dotted vertical line denotes the selected model with the best cross validation performance and the right dotted line the most parsimonious within the 1 standard error range of the performance from the best model (for reference purpose). (d) Balance of propensity scores before and after matching. (e)–(h) Those for closure as of April 10. (i) All and effective sample sizes and the maximum weight among the samples.

I also noticed that propensity scores computed for one of the subanalyses included, inverse-probability weighting, exhibited substantial/complete “separation” ( Heinze & Schemper, 2002) and most samples were essentially lost due to the substantial imbalance in the assigned weights ( Figure 4). Although separation of propensity scores can arise from overfitting, in this case it remained (while slightly ameliorated) even after addressing overfitting by Lasso regularisation ( Figure 5). This indicates that the treatment assignments may have been nearly deterministic in the dataset, which can compromise the performance of quasi-experimental causal inference via “positivity violation” ( Petersen *et al.*, 2020).

The authors did not use propensity scores in the Mahalanobis distance-based genetic matching for the main analysis as opposed to the general recommendation ( Diamond & Sekhon, 2013) (the authors cite King & Nielsen, 2019 as a reason not to use propensity scores, the authors of which however clarifies that their criticism does not apply to genetic matching). This means that the covariates that strongly determined the treatment assignment may not have received large weights (and therefore were not prioritised) in the matching process, which could leave unadjusted bias arising from these potential confounders. For example, many regression coefficients for prefecture dummy variables had large values (~5 or larger) in the Lasso-regularised model, whereas 236 out of 483 matched pairs of municipalities in the original analysis for April 6 were from different prefectures. The robustness to the above concerns could be assessed by computing ESS from another genetic matching including propensity scores and a calliper (to ensure the matched pairs have sufficiently similar features), which I report in the next section.

## Reanalysis with genetic matching with propensity scores and a calliper

**Figure 6.**
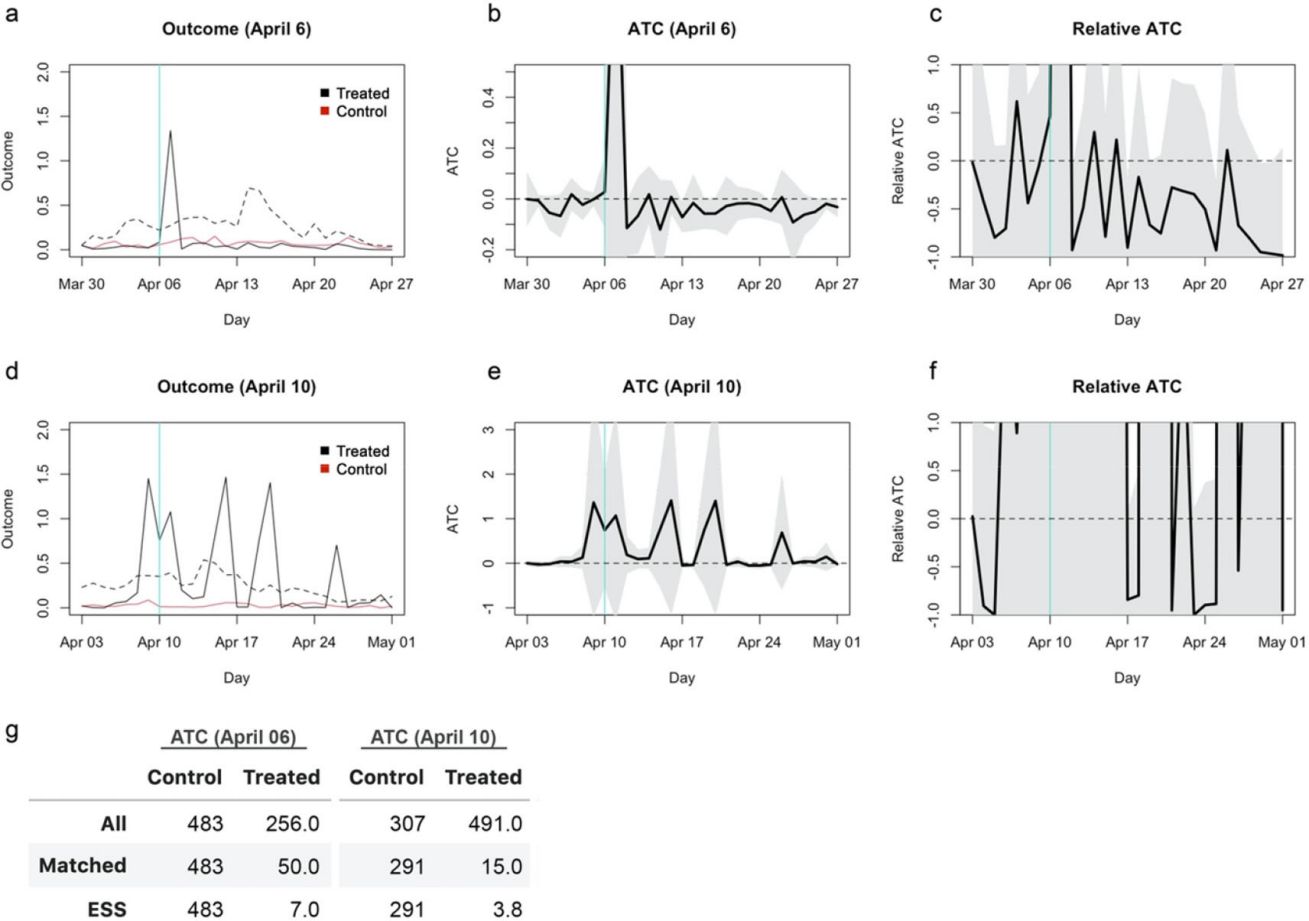
Re-estimated average treatment effect on the control (ATC) using a genetic matching with propensity scores and a calliper of 0.25. (a) The average outcome (incidence per capita) of the unmatched treatment (dashed black), matched treatment (solid black) and control (red) groups for closure as of April 6, 2020. (b) Absolute ATC estimates (black line) and 95% confidence intervals (shaded area) for closure as of April 6. (c) Relative ATC estimates and 95% confidence intervals for closure as of April 6. (d)–(f) Those for closure as of April 10.

I reanalysed the original dataset with the genetic matching algorithm incorporating propensity scores and a calliper and estimated ATCs for school closures as of Aril 6 and 10, 2020. Propensity scores were estimated by a Lasso-regularised linear regression model and included in genetic matching with a calliper of 0.25 ( Rosenbaum & Rubin, 1985). The results remained statistically insignificant and the confidence intervals for the relative effects covered most region from -100% to 100%, although the direction of the weak trend reversed for closure as of April 6 from the original study ( Figure 6). ESS of the matched treatment group was only 7 and 3.8 for April 6 and 10, respectively, indicating that the results relied on only a small set of samples that were repeatedly used in matching. Genetic matching is a generalisation of propensity score and Mahalanobis distance matching that searches for optimal covariate balance and thus should achieve no worse balance than matching using only Mahalanobis distance ( Diamond & Sekhon, 2013). The substantial loss of ESS in the updated genetic matching with propensity scores suggests that improved matching required more samples to be discarded and that both the original and current results are likely unreliable.

## Discussion and Conclusion

The reanalysis of Fukumoto *et al.* suggested that the study was inherently underpowered to identify the presence of causal effects of school closure on COVID-19. While I recognise the importance of their attempt to assess the school closure policy given its collateral effect imposed onto students and their families, I argue that their conclusion of “no causal effect” was not well supported by data due to the limited statistical power. Finding no mitigating effect itself would not be surprising as children were not the centre of the outbreak especially in the earliest phase ( Davies *et al.* 2020); nonetheless, evidence claiming “no effect” would need to show that effects were at least below the level of practical significance.

In addition to this issue of insufficient statistical power, which I demonstrated in the present reanalysis, two additional issues have been raised during the peer review process of this article. For one: the authors’ choice of ATC as the main estimand may have been suboptimal as Shiba has pointed out in his comment ( Shiba, 2022). The control group in the original study may have consisted of municipalities that did not need school closures because of low incidence. ATC in this context would represent the effect in settings where the policy was not needed, which is of limited political implication. To counterargue against school closures as a control policy, the authors should have aimed to robustly show insufficient effect of such a policy even in municipalities in which school closures had been a selectable option (possibly because of higher incidence rate, where an effective policy could be more impactful). For the other: residual confounding may have remained among the matched samples. Both ( Shiba, 2022) and ( Hayashi, 2022) expressed concern on the immediate positive effect on incidence rate (e.g. increased incidence) immediately after the implementation of school closures in the treated group, which Fukumoto et al. left unexplained. Unless a plausible causal mechanism in which school closures could increase COVID-19 incidence is provided, this gap between the treated and control group may indicate residual bias, which is unsurprising given my reanalysis results suggesting matching failure. Hayashi additionally suggested that the trend in incidence (e.g. increasing/decreasing) may be one of the potential confounding variables that had not been adjusted for in the original study ( Hayashi, 2022).

Altogether, these limitations represent difficulties in post-hoc causal analysis of mass interventions implemented without a built-in evaluation design such as randomisation. The fact that even the reasonably designed approach of Fukumoto *et al.* suffers insufficient power emphasises the importance of the “evidence-generating” philosophy in policy planning as has been promoted for medicine ( Embi & Payne, 2013).

## Data availability

### Underlying data

This study did not generate original data. The underlying dataset is available from the repository associated with the original study:

Harvard Dataverse. Replication Data for: No causal effect of school closures in Japan on the spread of COVID-19 in spring 2020. DOI: https://doi.org/10.7910/DVN/N803UQ ( Fukumoto *et al.* 2021a).

Data are available under the terms of the Creative Commons Zero “No rights reserved” data waiver.

### Extended data

Replication code along with the full analysis report (Extended data: Supplementary document) is available from a GitHub repository: https://github.com/akira-endo/reanalysis_Fukumoto2021.

Archived version of the above repository at time of publication is available from: Zenodo. akira-endo/reanalysis_Fukumoto2021: ‘Not finding causal effect’ is not ‘finding no causal effect’ of school closure on COVID-19. DOI: https://doi.org/10.5281/zenodo.6457916 ( Endo, 2022).

This project contains the following data:

- main.html/main.ipynb (Extended data: Supplementary document).
- replication codes and data from the original study ( Fukumoto *et al.* 2021a) which are partially modified and reused.
- replication codes for the analysis conducted in this study.

Data are available under the terms of the Creative Commons Attribution 4.0 International license (CC-BY 4.0).

## References

[ref8] Davies NG Klepac P Liu Y : Age-dependent effects in the transmission and control of COVID-19 epidemics. *Nat. Med.* 2020;26(26):1205–1211. 10.1038/s41591-020-0962-9 32546824

[ref5] Diamond A Sekhon JS : Genetic Matching for Estimating Causal Effects: A General Multivariate Matching Method for Achieving Balance in Observational Studies. *Rev. Econ. Stat.* 2013;95(3):932–945. 10.1162/REST_a_00318

[ref9] Embi PJ Payne PRO : Evidence Generating Medicine. *Med. Care.* 2013;51(8 Suppl 3):S87–S91. 10.1097/MLR.0b013e31829b1d66.51 23793052

[ref11] Endo A : akira-endo/reanalysis_Fukumoto2021: ’Not finding causal effect’ is not ’finding no causal effect’ of school closure on COVID-19 (v.1.0.0). *Zenodo.* 2022. 10.5281/zenodo.6457916 PMC11401980 39281333

[ref1] Fukumoto K McClean CT Nakagawa K : No causal effect of school closures in Japan on the spread of COVID-19 in spring 2020. *Nat. Med.* 2021a;27:2111–2119. 10.1038/s41591-021-01571-8 34707318 PMC8674136

[ref10] Fukumoto K McClean CT Nakagawa K : Replication data for: no causal effect of school closures in Japan on the spread of COVID-19 in spring 2020. *Harvard Dataverse.* 2021b;27:2111–2119. (Accessed: 8 November 2021). 10.7910/DVN/N803UQ PMC8674136 34707318

[ref12] Hayashi TI : Peer Review Report For: ‘Not finding causal effect’ is not ‘finding no causal effect’ of school closure on COVID-19 [version 1; peer review: 1 approved, 1 approved with reservations]. *F1000Research.* 2022;11:456. 10.5256/f1000research.123641.r136221 PMC11401980 39281333

[ref3] Heinze G Schemper M : A solution to the problem of separation in logistic regression. *Stat. Med.* 2002;21:2409–2419. 10.1002/sim.1047 12210625

[ref13] King G Lucas C Nielsen R : The Balance-Sample Size Frontier in Matching Methods for Causal Inference. *Am. J. Political Sci.* 2017;61(2):473–489. 10.1111/ajps.12272

[ref6] King G Nielsen R : Why propensity scores should not be used for matching. *Polit. Anal.* 2019;27(4):435–454. 10.1017/pan.2019.11

[ref4] Petersen ML Porter KE Gruber S : Diagnosing and responding to violations in the positivity assumption. *Stat. Methods Med. Res.* 2020;21(1):31–54. 10.1177/0962280210386207 21030422 PMC4107929

[ref7] Rosenbaum PR Rubin DB : Constructing a Control Group Using Multivariate Matched Sampling Methods That Incorporate the Propensity Score. *Am. Stat.* 1985;39(1):33–38. 10.1080/00031305.1985.10479383

[ref14] Shook-Sa BE Hudgens MG : Power and sample size for observational studies of point exposure effects. *Biometrics.* 2022 Mar;78(1):388–398. 10.1111/biom.13405 33226116 PMC8141060

[ref15] Shiba K : Peer Review Report For: ‘Not finding causal effect’ is not ‘finding no causal effect’ of school closure on COVID-19 [version 1; peer review: 1 approved, 1 approved with reservations]. *F1000Research.* 2022;11:456. 10.5256/f1000research.123641.r136223 PMC11401980 39281333

[ref2] Viner RM Russell SJ Croker H : School closure and management practices during coronavirus outbreaks including COVID-19: a rapid systematic review. *Lancet Child Adolesc. Health.* 2020;4(5):397–404. 10.1016/S2352-4642(20)30095-X 32272089 PMC7270629

