# Supplementary material for: ‘Not finding causal effect’ is not ‘finding no causal effect’ of school closure on COVID-19
Source: F1000Res. 2024 Apr 16;11:456. Originally published 2022 Apr 25. [Version 2] doi: 10.12688/f1000research.111915.2 (PMC11401980; doi:10.12688/f1000research.111915.2)
Supplement: Peer review history

# Reviewer report: Reviewer response for version 2

**DOI:** 10.5256/f1000research.140839.r301839

**Published:** 2024-09-14

**Authors:** Eiji Yamamura

**Affiliations:** 1 Seinan Gakuin University, Fukuoka, Japan

Report on 

 ‘Not finding causal effect’ is not ‘finding no causal effect’ of school closure on COVID-19

 The aim of the study is to examine whether PSM (Propensity Score Matching) method of Fukumoto, McClean, Nakagawa (FMN 2021) is appropriately conducted.  Main finding of this study is that ESS has substantially reduced due to imbalanced covariates between samples. FMN did not find causal effect of school closure on incidence rates in the early phase of COVID-19 pandemic. However, actually, their estimation results are not reliable because of insufficient EES.  In my view, validity of set of covariates to calculate propensity score and its results in the “first” stage have not been scrutinized and discussed profoundly. However, other reports have already pointed out. The author has already replied to it. The focus of this study seems to be on balance issue and EES. I agree with it because this study is correspondence rather than full paper.

 Here, I raise only several issues remain to be addressed.

 Major issues:

 1. For obtaining results of Fig.6, the author conducted estimation to set the caliper to be 0.25 by following the classical study of Rosenbaum & Rubin (1985) as below.

 "Propensity scores were estimated by a Lasso-regularised linear regression model and included in genetic matching with a calliper of 0.25 (Rosenbaum & Rubin, 1985)".

 2. A caliper of 0.25 is widely used for matching in empirical studies. However, more recent works scrutinize the optimal value of calipers. For instance, Austin (2011) recommend that researchers match the propensity score using calipers being equal to 0.2. For illustrating Fig 6, author should use 0.2 rather than 0.25.  Otherwise, author should justify his choosing 0.25 as caliper based on recent literatures.

 References.

 Austin PC, 2011 (Ref 1)

 Minor issues:

 1. This study rigorously scrutinized the validity of PSM. In conclusion, the author derived more general argument than what has been done in this study. PSM has been widely used in empirical studies. However, in my view, most of studies using PSM mainly reported the main results, while not having sufficiently examined its validity. Actually, I believe that PSM results reported in many studies published in peer review journals would not meet the criteria to justify them (not valid) if researchers rigorously test it. FMN is one of them. Therefore, it seems better to narrow down the points to limitation of PSM.

 2. I found inconsistency between Figs and its explanation in main body of text. Further, there seems to be several errors in Figs. As I read the text, referring to Figs, I became confused. Careless mistakes in basic information should be corrected to avoid reader’s misunderstanding.

 (1) In caption of Fig 1, (d) appeared two times. “(d) Relative ATT for the closure as of April 6, 2020. (d) Comparison of sample sizes.” This should be “(c) Relative ATT for the closure as of April 6, 2020. (d) Comparison of sample sizes.”  

 (2) Concerning Fig 1, I cannot find “Relative ATT (April 10)” although “Relative ATT (April 06)” was presented.  It is strange because readers can compare results between different setting in Figures 2-6. Author should present “Relative ATT (April 10)” as Fig 1 d (The current Fig 1 d should be “Fig 1 e”), otherwise explain the reason not to indicated it.

 (3) In caption of Figs 2. Tittle of Fig 2a is "Outcome (April 6): 50% mitigating effect" wile that of Fig 2 d is “Outcome (April 6): 80% mitigating effect". From these titles and contents of Figs 2, I believe that Fig 2a, b and c are results of "Outcome (April 6): 50% mitigating effect" while Fig 2d, e and f "Outcome (April 6): 80% mitigating effect".

 "ATT for the 80% mitigating effect was also statistically insignificant (Figure 2c and 2d),"

 Probably, the second sentence should be “ATT for the 80% mitigating effect was also statistically insignificant (Figure 2e and 2f),”

 (4) In the end of Caption of Fig 2, I found “(d)–(f) Those for closure as of April 10”.

 The sentence should be “In (d)–(f), 80% mitigating effects”. Probably, the author copied the caption of Fig 6 although caption of Fig 6 is correct.

 (5) Comment (4) also applied to Figs 3.

Are arguments sufficiently supported by evidence from the published literature or by new data and results?

Yes

Is the conclusion balanced and justified on the basis of the presented arguments?

Yes

Is the rationale for commenting on the previous publication clearly described?

Yes

Are any opinions stated well-argued, clear and cogent?

Yes

Reviewer Expertise:

Behavioral Economics, Applied Economics

I confirm that I have read this submission and believe that I have an appropriate level of expertise to confirm that it is of an acceptable scientific standard, however I have significant reservations, as outlined above.

## References

[1] : Optimal caliper widths for propensity-score matching when estimating differences in means and differences in proportions in observational studies. *Pharm Stat* .2011;10(2) : 10.1002/pst.433 150-61 10.1002/pst.433 20925139 PMC3120982

# Reviewer report: Reviewer response for version 1

**DOI:** 10.5256/f1000research.123641.r136221

**Published:** 2022-07-04

**Authors:** Takehiko I. Hayashi

**Affiliations:** 1 Social Systems Division, National Institute for Environmental Studies, Tsukuba, Japan

This article reanalyzed Fukumoto *et al. *(2021), which concluded that school closures had no causal effect on the spread of COVID-19 in Spring 2020 in Japan. The author first examined the robustness of the conclusion of Fukumoto *et al.* (2021) to the way the indicator is set up. The author then conducted simulations of cases with hypothetical 50% or 80% mitigating effects using the same study design and sample size as Fukumoto *et al.* (2021). As I state below (see Major points), I agree that these results (Figs. 1 and 2) support the author's main argument that Fukumoto *et al.* (2021) did not provide sufficient statistical power to conclude there was “no causal effect.” The author also pointed out the risk of positivity violation and the resulting small effective sample size in propensity score analysis (Figs. 4 and 5). I believe this issue should be further examined because there was a possibly nonnegligible level of residual confounding (see Minor points), but the additional examination may be beyond the scope of this article. Overall, I agree that the authors' arguments are adequately supported by the results presented. In general, the issue of statistical power tends to receive less focus than the issue of identification in the practice of causal inference. However, statistical power is always important when discussing the effects of policies in real-world contexts. This paper is a good practice example to remind us of the fundamentals of statistical inference.

**Major points:**

 The primary purpose of this study is to examine the statistical power of the analysis of Fukumoto *et al. *(2021). Although Fukumoto *et al.* (2021) is an elaborate study that examined most possible considerations, it lacks an examination of statistical power, and statistical power is a logically essential issue if one is to conclude “no causal effect” based on the lack of statistical significance.

 The author first addressed the issue of outcome measures. I agree with the author that effect estimates relative to the baseline incidence may be superior to per-capita incidence rates as an outcome measure. As the author stated, taking only non-negative values for the incidence rate can be a large problem when the incidence rate of the control population is very low, as it was in this case. I think that the spikes in the red line of the control population in Figures 1c and 1i of Fukumoto *et al.* (2021) (which diverge from the black line of the matched treatment population, indicating a failure to construct an adequate counterfactual) also suggest a disadvantage of using per capita rates, given the explanation that these spikes were caused by the small sizes of the focal municipalities (see the Supplementary Information of Fukumoto *et al.* 2021, P. 11, Lines 3–11). Although I do not immediately conclude that the advantage of using effect estimates relative to the baseline incidence as the indicator is absolute, using this indicator is one of the possible reasonable choices. The results using this indicator (Fig. 1) showed an inherent lack of power in the analysis, illustrating that the conclusions of Fukumoto *et al.* (2021) (implicitly assuming a degree of statistical power of the analyses) are not robust to the way the indicator is set up.

 The author's next approach is more direct. The author conducted simulations of cases with hypothetical 50% or 80% mitigating effects using the same study design and sample size as Fukumoto *et al.* (2021). The simulation showed that statistical significance was hardly detected even for substantial effects (Fig. 2). I believe these results convincingly demonstrate that the design and data of Fukumoto et al. (2021) did not provide sufficient statistical power to conclude “no causal effect.”

 The above results (Figs. 1 and 2) logically support the author's main argument that Fukumoto *et al.* (2021) did not provide sufficient statistical power to conclude "no causal effect."

**Minor points:**

 Although the following comments may address issues that are beyond the scope of this study, the issues themselves are essential. These comments are intended as suggestions and not mandatory revisions of this article.

 First, as the other reviewer (Dr. Shiba) stated, the validity of the estimand is essential. I agree that ATT should have been the main estimand if Fukumoto *et al.* (2021) mention the efficacy of the policy in the real-world context. I recommend that the author discuss this point further.

 Second, I think more consideration needs to be given to the possibility of insufficient adjustment (i.e., residual confounding). The author's mention of the risk of positivity violation and the resulting small effective sample size in propensity score analysis is a good point (Figs. 4 and 5). The separation of the propensity score distribution implies the inherent difficulties in matching important factors, especially those having a large effect on both treatment and outcome, which can introduce confounding. This is a real concern because many major covariates were not sufficiently adjusted. A well-known recommendation for an acceptable degree of ASMD after matching is ≤0.1 (Nguyen et al. 2017). However, the ASMDs of many covariates were actually ≥0.2 in this case (Table S3 in the Supplementary Information of Fukumoto et al. 2021). In general, I think it is difficult to state that “differences between the matched groups cannot be attributed to previous levels of infection or any other covariates” when the absolute value of ASMD was ≥0.2 in many covariates. I recommend the author check the loveplot of the matching of April 6, 10, and some cases for ATTs with the reference dashed line at 0.1 ASMD. I also recommend presenting the importance of covariates (e.g., specifying covariates having high standardized coefficient values in the propensity score estimation with red symbols) in the loveplots (see the Note below concerning the need for a loveplot).

 Third, discussing the possibility of missing important covariates may also be worthwhile. Some unexpected behavior of the Fukumoto *et al.* (2021) data suggests the possibility of residual confounding (due to the lack of incorporation of important covariates). For example, in Figure 1g of Fukumoto *et al. *(2021), large (see the absolute values) spikes appear only in the matching treated municipalities (and no spikes appear in all treated municipalities). In general, matching is expected to reduce the difference between treated and untreated baselines. Thus, it seems difficult to naturally explain the occurrence of large spikes only in the matched municipalities (unless the treatment actually increased outcomes or the effective sample size is very small). It is possible that important covariates were missed. For example, the trends (not sum) of the incidence rate before treatment was not included as covariates, but they might cause such spikes as follows. In the matching process, matched municipalities tend to have a similar value of the sum of the 7-day incidence rate. Here, the same value of this sum (e.g., 100 incidents per unit) can arise from municipalities that have different time trends (i.e., both increasing and decreasing trends are possible). In this situation, if the treatment (school closure) decision-making was affected by the increasing/decreasing trend, the treated group may tend to include municipalities (with 100 incidents per unit before 7 days) when there is an increasing trend. Similarly, the untreated group may tend to include municipalities (with 100 incidents per unit before 7 days) when there is a decreasing trend. In this case, the spikes (i.e., the difference in post-treatment outcomes) only in matched treated municipalities (as in Fig. 1g of Fukumoto *et al*., 2021) could occur as an artifact of the inertia of the temporal trend (not sum) from the preceding 7 days.

*Note on the need for a loveplot:* The following description in Fukumoto *et al.* (2021, P. 2114) is not sufficiently correct in two points: “Moreover, the differences in other covariates between the treated and control groups were also much smaller after matching than before (Supplementary Fig. 1 and Supplementary Table 3). Therefore, differences between the matched groups cannot be attributed to previous levels of infection or any other covariates.” First, whether confounding was removed or not does not depend on the *relative* ratio before/after ASMD; rather, it depends on the *absolute* magnitude of ASMD. Even if ASMD becomes relatively much smaller after matching, if the absolute magnitude of ASMD was over 0.2 in many covariates, it is difficult to state that “differences between the matched groups cannot be attributed to previous levels of infection or any other covariates” in a general sense. Second, a smaller *average* ASMD of many covariates does not assure the removal of confounding. The removal of confounding needs to balance *important covariates* satisfying a backdoor criterion (not an *average* of all covariates). Practically, we can speculate about the importance of covariates from the effects of these covariates on treatments and outcomes (c.f., VanderWeele 2019). Figure S1 in the Supplementary Information of Fukumoto *et al.* (2021) did not provide good information with which to judge these two essential points in terms of the reduction of confounding. To make such a judgment, a loveplot with a reference line at 0.1 is suitable and is also a standard practice.

Are arguments sufficiently supported by evidence from the published literature or by new data and results?

Yes

Is the conclusion balanced and justified on the basis of the presented arguments?

Yes

Is the rationale for commenting on the previous publication clearly described?

Yes

Are any opinions stated well-argued, clear and cogent?

Yes

Reviewer Expertise:

Risk analysis, statistical causal inference, and environmental data analyis

I confirm that I have read this submission and believe that I have an appropriate level of expertise to confirm that it is of an acceptable scientific standard.

## References

[1] : Double-adjustment in propensity score matching analysis: choosing a threshold for considering residual imbalance. *BMC Med Res Methodol* .2017;17(1) : 10.1186/s12874-017-0338-0 78 10.1186/s12874-017-0338-0 28454568 PMC5408373

[2] : Principles of confounder selection. *Eur J Epidemiol* .2019;34(3) : 10.1007/s10654-019-00494-6 211-219 10.1007/s10654-019-00494-6 30840181 PMC6447501

# Response

**Published:** 2024-04-09

**Authors:** Akira Endo

**Affiliations:** London School of Hygiene & Tropical Medicine, UK

***> I thank the reviewers for their constructive feedback. While I regret the extended time it took to revise the manuscript in response to their comments, I believe the revised version and the following responses will address the reviewers' concerns. Specifically, I added two key discussion points that both reviewers agreed should be included: (i) choice of the estimand (ATC vs ATT) and (ii) potential residual confounding.***

 This article reanalyzed Fukumoto *et al. *(2021), which concluded that school closures had no causal effect on the spread of COVID-19 in Spring 2020 in Japan. The author first examined the robustness of the conclusion of Fukumoto *et al.* (2021) to the way the indicator is set up. The author then conducted simulations of cases with hypothetical 50% or 80% mitigating effects using the same study design and sample size as Fukumoto *et al.* (2021). As I state below (see Major points), I agree that these results (Figs. 1 and 2) support the author's main argument that Fukumoto *et al.* (2021) did not provide sufficient statistical power to conclude there was “no causal effect.” The author also pointed out the risk of positivity violation and the resulting small effective sample size in propensity score analysis (Figs. 4 and 5). I believe this issue should be further examined because there was a possibly nonnegligible level of residual confounding (see Minor points), but the additional examination may be beyond the scope of this article. Overall, I agree that the authors' arguments are adequately supported by the results presented. In general, the issue of statistical power tends to receive less focus than the issue of identification in the practice of causal inference. However, statistical power is always important when discussing the effects of policies in real-world contexts. This paper is a good practice example to remind us of the fundamentals of statistical inference.

***> I thank the reviewer for supporting the points discussed in the manuscript and for offering additional comments on its content.***

**Major points:**

 The primary purpose of this study is to examine the statistical power of the analysis of Fukumoto *et al. *(2021). Although Fukumoto *et al.* (2021) is an elaborate study that examined most possible considerations, it lacks an examination of statistical power, and statistical power is a logically essential issue if one is to conclude “no causal effect” based on the lack of statistical significance.

 The author first addressed the issue of outcome measures. I agree with the author that effect estimates relative to the baseline incidence may be superior to per-capita incidence rates as an outcome measure. As the author stated, taking only non-negative values for the incidence rate can be a large problem when the incidence rate of the control population is very low, as it was in this case. I think that the spikes in the red line of the control population in Figures 1c and 1i of Fukumoto *et al.* (2021) (which diverge from the black line of the matched treatment population, indicating a failure to construct an adequate counterfactual) also suggest a disadvantage of using per capita rates, given the explanation that these spikes were caused by the small sizes of the focal municipalities (see the Supplementary Information of Fukumoto *et al.* 2021, P. 11, Lines 3–11). Although I do not immediately conclude that the advantage of using effect estimates relative to the baseline incidence as the indicator is absolute, using this indicator is one of the possible reasonable choices. The results using this indicator (Fig. 1) showed an inherent lack of power in the analysis, illustrating that the conclusions of Fukumoto *et al.* (2021) (implicitly assuming a degree of statistical power of the analyses) are not robust to the way the indicator is set up.

 The author's next approach is more direct. The author conducted simulations of cases with hypothetical 50% or 80% mitigating effects using the same study design and sample size as Fukumoto *et al.* (2021). The simulation showed that statistical significance was hardly detected even for substantial effects (Fig. 2). I believe these results convincingly demonstrate that the design and data of Fukumoto et al. (2021) did not provide sufficient statistical power to conclude “no causal effect.”

 The above results (Figs. 1 and 2) logically support the author's main argument that Fukumoto *et al.* (2021) did not provide sufficient statistical power to conclude "no causal effect."

***> I thank the reviewer for appreciating and further discussing in detail the main points of my criticism. Regarding the choice of indicator, in response to Reviewer 1 (Dr Shiba)’s comment, I have included additional reasoning why relative effect is more relevant to this infectious disease context.***

**Minor points:**

 Although the following comments may address issues that are beyond the scope of this study, the issues themselves are essential. These comments are intended as suggestions and not mandatory revisions of this article.

 First, as the other reviewer (Dr. Shiba) stated, the validity of the estimand is essential. I agree that ATT should have been the main estimand if Fukumoto *et al.* (2021) mention the efficacy of the policy in the real-world context. I recommend that the author discuss this point further.

***> Discussion on the choice of estimand (along with that on residual confounding) has been included in the Discussion and Conclusion section. Please also see the response to Reviewer 1.***

 Second, I think more consideration needs to be given to the possibility of insufficient adjustment (i.e., residual confounding). The author's mention of the risk of positivity violation and the resulting small effective sample size in propensity score analysis is a good point (Figs. 4 and 5). The separation of the propensity score distribution implies the inherent difficulties in matching important factors, especially those having a large effect on both treatment and outcome, which can introduce confounding. This is a real concern because many major covariates were not sufficiently adjusted. A well-known recommendation for an acceptable degree of ASMD after matching is ≤0.1 (Nguyen et al. 2017). However, the ASMDs of many covariates were actually ≥0.2 in this case (Table S3 in the Supplementary Information of Fukumoto et al. 2021). In general, I think it is difficult to state that “differences between the matched groups cannot be attributed to previous levels of infection or any other covariates” when the absolute value of ASMD was ≥0.2 in many covariates. I recommend the author check the loveplot of the matching of April 6, 10, and some cases for ATTs with the reference dashed line at 0.1 ASMD. I also recommend presenting the importance of covariates (e.g., specifying covariates having high standardized coefficient values in the propensity score estimation with red symbols) in the loveplots (see the Note below concerning the need for a loveplot).

***> I thank the reviewer for constructive suggestion to further investigate the appropriateness of matching in the original study. While I agree that a loveplot would provide more in-depth understanding of what might have gone wrong with the original analysis, the aim of my article is to highlight the existence of the issue (not necessarily revealing every detail of the individual issues), which I believe has already been demonstrated. Once the potential issue is identified as such, in principle the original authors should be responsible for conducting robust analysis to defend their findings.***

 Third, discussing the possibility of missing important covariates may also be worthwhile. Some unexpected behavior of the Fukumoto *et al.* (2021) data suggests the possibility of residual confounding (due to the lack of incorporation of important covariates). For example, in Figure 1g of Fukumoto *et al. *(2021), large (see the absolute values) spikes appear only in the matching treated municipalities (and no spikes appear in all treated municipalities). In general, matching is expected to reduce the difference between treated and untreated baselines. Thus, it seems difficult to naturally explain the occurrence of large spikes only in the matched municipalities (unless the treatment actually increased outcomes or the effective sample size is very small). It is possible that important covariates were missed. For example, the trends (not sum) of the incidence rate before treatment was not included as covariates, but they might cause such spikes as follows. In the matching process, matched municipalities tend to have a similar value of the sum of the 7-day incidence rate. Here, the same value of this sum (e.g., 100 incidents per unit) can arise from municipalities that have different time trends (i.e., both increasing and decreasing trends are possible). In this situation, if the treatment (school closure) decision-making was affected by the increasing/decreasing trend, the treated group may tend to include municipalities (with 100 incidents per unit before 7 days) when there is an increasing trend. Similarly, the untreated group may tend to include municipalities (with 100 incidents per unit before 7 days) when there is a decreasing trend. In this case, the spikes (i.e., the difference in post-treatment outcomes) only in matched treated municipalities (as in Fig. 1g of Fukumoto *et al*., 2021) could occur as an artifact of the inertia of the temporal trend (not sum) from the preceding 7 days.

**> Discussion on possible residual confounder has been included in the Discussion and Conclusion section. Please also see the response to Reviewer 1.**

*Note on the need for a loveplot:* The following description in Fukumoto *et al.* (2021, P. 2114) is not sufficiently correct in two points: “Moreover, the differences in other covariates between the treated and control groups were also much smaller after matching than before (Supplementary Fig. 1 and Supplementary Table 3). Therefore, differences between the matched groups cannot be attributed to previous levels of infection or any other covariates.” First, whether confounding was removed or not does not depend on the *relative* ratio before/after ASMD; rather, it depends on the *absolute* magnitude of ASMD. Even if ASMD becomes relatively much smaller after matching, if the absolute magnitude of ASMD was over 0.2 in many covariates, it is difficult to state that “differences between the matched groups cannot be attributed to previous levels of infection or any other covariates” in a general sense. Second, a smaller *average* ASMD of many covariates does not assure the removal of confounding. The removal of confounding needs to balance *important covariates* satisfying a backdoor criterion (not an *average* of all covariates). Practically, we can speculate about the importance of covariates from the effects of these covariates on treatments and outcomes (c.f., VanderWeele 2019). Figure S1 in the Supplementary Information of Fukumoto *et al.* (2021) did not provide good information with which to judge these two essential points in terms of the reduction of confounding. To make such a judgment, a loveplot with a reference line at 0.1 is suitable and is also a standard practice.

# Reviewer report: Reviewer response for version 1

**DOI:** 10.5256/f1000research.123641.r136223

**Published:** 2022-04-29

**Authors:** Koichiro Shiba

**Affiliations:** 1 Department of Epidemiology, Harvard T.H. Chan School of Public Health, Boston, MA, USA

This article provides a critical re-assessment of the recent paper that concluded that school closures had no causal effect on the spread of COVID-19 in Spring 2020 (Fukumoto *et al.*, 2021). The author of this article argued that the original analysis (and the refined version presented in the current article) was likely underpowered and unreliable. The author raised a great point about the potential violation of positivity, about which the original paper provided little discussion. I appreciate the author for bringing our attention to these important issues and allowing me to engage in carefully reading the original article. I agree with the author that it is vital to be mindful that the absence of “evidence” defined by the lack of statistical significance is not evidence of absence. Hence, I find this type of article assessing alternative explanations for the null findings in the original paper particularly valuable. 

 I provide some major and minor suggestions to strengthen the current manuscript further.

**Major points:**

 The manuscript is currently written as if the lack of statistical power is the primary (and perhaps only) issue that might explain the original paper’s null findings. The author does discuss the possibility of unadjusted confounding later in the paper, but the way the Introduction and the Conclusion were written made it seem a secondary problem. I suggest the author provide a more in-depth discussion on other potential issues. There are at least two other issues I think are worth discussing: the choice of causal estimand and residual confounding. 

 To me, the most significant limitation of the original article is its focus on identifying and estimating ATC. The controls in the study were the municipalities that did not (need to) enforce school closures in the Spring of 2020; that is, they were most likely the areas that do not benefit from school closures because they were not experiencing the spread of COVID-19 to begin with, which is supported by the extremely low (nearly zero) confirmed cases in the control group shown in Figure 1. ATC, in this context, is of little policy relevance because such municipalities would have few cases regardless of the implementation of the school closures (the counterfactual “what would happen had they closed the schools” would not differ much from the reality). The null finding for ATC is somewhat expected. What we want to know instead is if the spread in the treated (i.e., municipalities that were experiencing the rise in confirmed cases and had to decide to close schools) would have been worse without the school closures (i.e., ATT). I know that the original article did investigate ATT as a sensitivity analysis, but it was based on the data stemming from the school closure on only one date (April 6) with a much smaller matched sample size (as the treated was smaller in number). They did not provide key supplementary information for the ATT analysis (e.g., covariate balance after matching) either. I understand that, with the available data, ATC (versus ATT) was easier to estimate as there were more controls; but that does not justify their oversimplified conclusion that there is no effect of school closures in Japan because the effect of a hypothetical intervention can vary substantially depending on the target populations. 

 The original article and the current article took a careful approach to mitigating bias due to confounding; yet, I see some evidence of residual confounding. The two articles indicate that ATC immediately after the school closures was positive point-estimate-wise, at least for some dates. The original article's authors wrote: “The ATC values suggest that municipalities that closed their schools mostly increased the number of cases”. If matching was successful and there was no unadjusted confounding, this statement would be true. Yet, they provide no compelling explanation for why school closures may causally “increase” confirmed cases. I cannot think of any. If not causal, the increase in cases among the treated municipalities in the matched sample is likely due to residual confounding—they had a reason to be concerned about the spread and decided to close the school, which was not captured by the observed covariates, including the prior outcome values and school closure status. The author of the current article raised an excellent point regarding residual bias, but some additional discussion on this issue would be appreciated.

**Minor points:**

1. Page 3: “Moreover, matching technique used to minimise confounding has a known side effect of...” needs citation.
2. Page 3: “The effect estimates relative to the baseline incidence would be a more intuitive and interpretable measure for assessment of its practical use.” This needs more justification. The additive effect measure has its own advantage because it can speak directly to the population impacts of the intervention.
3. Page 3: The sentence starting, “However, the theoretical ground is unclear...” seems incomplete.
4. Page 3: “ATC and average treatment effect on the treatment: ATT) and their confidence intervals relative to the average outcome (incidence rate per capita) in the control group (Figure 1).” This sentence seems incomplete. Perhaps delete the part after “relative to...“.
5. Page 3: Spell out ESS and provide a bit more context of what it is.

Are arguments sufficiently supported by evidence from the published literature or by new data and results?

Yes

Is the conclusion balanced and justified on the basis of the presented arguments?

Partly

Is the rationale for commenting on the previous publication clearly described?

Yes

Are any opinions stated well-argued, clear and cogent?

Partly

Reviewer Expertise:

Public health, epidemiology, causal inference

I confirm that I have read this submission and believe that I have an appropriate level of expertise to confirm that it is of an acceptable scientific standard, however I have significant reservations, as outlined above.

# Response

**Published:** 2024-04-09

**Authors:** Akira Endo

**Affiliations:** London School of Hygiene & Tropical Medicine, UK

***> I thank the reviewers for their constructive feedback. While I regret the extended time it took to revise the manuscript in response to their comments, I believe the revised version and the following responses will address the reviewers' concerns. Specifically, I added two key discussion points that both reviewers agreed should be included: (i) choice of the estimand (ATC vs ATT) and (ii) potential residual confounding.***

 This article provides a critical re-assessment of the recent paper that concluded that school closures had no causal effect on the spread of COVID-19 in Spring 2020 (Fukumoto *et al.*, 2021). The author of this article argued that the original analysis (and the refined version presented in the current article) was likely underpowered and unreliable. The author raised a great point about the potential violation of positivity, about which the original paper provided little discussion. I appreciate the author for bringing our attention to these important issues and allowing me to engage in carefully reading the original article. I agree with the author that it is vital to be mindful that the absence of “evidence” defined by the lack of statistical significance is not evidence of absence. Hence, I find this type of article assessing alternative explanations for the null findings in the original paper particularly valuable. 

 I provide some major and minor suggestions to strengthen the current manuscript further.

***> I thank the reviewer for appreciating my criticism and providing useful suggestions.***

**Major points:**

 The manuscript is currently written as if the lack of statistical power is the primary (and perhaps only) issue that might explain the original paper’s null findings. The author does discuss the possibility of unadjusted confounding later in the paper, but the way the Introduction and the Conclusion were written made it seem a secondary problem. I suggest the author provide a more in-depth discussion on other potential issues. There are at least two other issues I think are worth discussing: the choice of causal estimand and residual confounding. 

 To me, the most significant limitation of the original article is its focus on identifying and estimating ATC. The controls in the study were the municipalities that did not (need to) enforce school closures in the Spring of 2020; that is, they were most likely the areas that do not benefit from school closures because they were not experiencing the spread of COVID-19 to begin with, which is supported by the extremely low (nearly zero) confirmed cases in the control group shown in Figure 1. ATC, in this context, is of little policy relevance because such municipalities would have few cases regardless of the implementation of the school closures (the counterfactual “what would happen had they closed the schools” would not differ much from the reality). The null finding for ATC is somewhat expected. What we want to know instead is if the spread in the treated (i.e., municipalities that were experiencing the rise in confirmed cases and had to decide to close schools) would have been worse without the school closures (i.e., ATT). I know that the original article did investigate ATT as a sensitivity analysis, but it was based on the data stemming from the school closure on only one date (April 6) with a much smaller matched sample size (as the treated was smaller in number). They did not provide key supplementary information for the ATT analysis (e.g., covariate balance after matching) either. I understand that, with the available data, ATC (versus ATT) was easier to estimate as there were more controls; but that does not justify their oversimplified conclusion that there is no effect of school closures in Japan because the effect of a hypothetical intervention can vary substantially depending on the target populations. 

 The original article and the current article took a careful approach to mitigating bias due to confounding; yet, I see some evidence of residual confounding. The two articles indicate that ATC immediately after the school closures was positive point-estimate-wise, at least for some dates. The original article's authors wrote: “The ATC values suggest that municipalities that closed their schools mostly increased the number of cases”. If matching was successful and there was no unadjusted confounding, this statement would be true. Yet, they provide no compelling explanation for why school closures may causally “increase” confirmed cases. I cannot think of any. If not causal, the increase in cases among the treated municipalities in the matched sample is likely due to residual confounding—they had a reason to be concerned about the spread and decided to close the school, which was not captured by the observed covariates, including the prior outcome values and school closure status. The author of the current article raised an excellent point regarding residual bias, but some additional discussion on this issue would be appreciated.

***> I thank the reviewer for pointing out the existence of potential issues that I did not emphasise in the paper. I believe these additional issues are indeed worth mentioning in the manuscript. Meanwhile, I would prefer to retain the lack of statistical power as the primary issue because the present manuscript leverages the results of re-analysis focusing on the statistical power. Moreover, the two additional points suggested would also eventually come down to the problem of limited sample size and reporting results without considering the statistical power / effective size. For example, as the reviewer suggests, the choice of ATC was probably not ideal in the original study’s context because of low incidence levels in the control group in the first place. However, the same study design focusing on ATC could still have found an effect (if there is a true effect and) if the sample size (in this case both the number of included municipalities and the number of cases reported in these municipalities) was sufficient.***

***Instead, I would like to propose changing the previous Conclusion section to the “Discussion and Conclusion” section and citing reviewers’ reports to discuss the suggested two points there. This allows me to separate the criticisms derived from my own analysis from those that were not, and also to appropriately acknowledge that the ideas came from the reviewers’ suggestions. I am aware that it may be a rather unusual practice in academic publications; however, given the nature of the publishing model of F1000 with citable open reviews and the fact that the reviewers provided new discussion points that were absent from the original version, I would like to opt for offering credit to the reviewers who contributed their time for the scholarly discussion.***

**(Added to Discussion and Conclusion section):**

**In addition to this issue of insufficient statistical power, which I demonstrated in the present reanalysis, two additional issues have been raised during the peer review process of this article. For one: the authors’ choice of ATC as the main estimand may have been suboptimal as Shiba has pointed out in his comment (Shiba, 2022). The control group in the original study may have consisted of municipalities that did not need school closures because of low incidence. ATC in this context would represent the effect in settings where the policy was not needed, which is of limited political implication. To counterargue against school closures as a control policy, the authors should have aimed to robustly show insufficient effect of such a policy even in municipalities in which school closures had been a selectable option (possibly because of higher incidence rate, where an effective policy could be more impactful). For the other: residual confounding may have remained among the matched samples. Both (Shiba, 2022) and (Hayashi, 2022) expressed concern on the immediate positive effect on incidence rate (e.g. increased incidence) immediately after the implementation of school closures in the treated group, which Fukumoto et al. left unexplained. Unless a plausible causal mechanism in which school closures could increase COVID-19 incidence is provided, this gap between the treated and control group may indicate residual bias, which is unsurprising given my reanalysis results suggesting matching failure. Hayashi additionally suggested that the trend in incidence (e.g. increasing/decreasing) may be one of the potential confounding variables that had not been adjusted for in the original study (Hayashi, 2022).**

**Minor points:**

1. Page 3: “Moreover, matching technique used to minimise confounding has a known side effect of...” needs citation.

***> We have newly cited King et al. (2017).***

  

1. Page 3: “The effect estimates relative to the baseline incidence would be a more intuitive and interpretable measure for assessment of its practical use.” This needs more justification. The additive effect measure has its own advantage because it can speak directly to the population impacts of the intervention.

***> We have added an explanation that the relative risk reduction is particularly relevant because of the dynamic nature of the infectious disease transmission:***

**Infectious disease risks are inherently dynamic; more current infections in a population would result in a greater risk of infection among susceptible individuals through increased encounters with infectious others. This means that the effect of school closures, which intended to reduce contacts at schools, should also depend on the baseline incidence in the population because the risk of infection averted would be the reduction in contacts multiplied by the probability that the contacts were otherwise with infectious individuals.**


* *

1. Page 3: The sentence starting, “However, the theoretical ground is unclear...” seems incomplete.

***> I have replaced “to” with “on” but believe the sentence itself is complete (“theoretical ground is unclear”).***

  

1. Page 3: “ATC and average treatment effect on the treatment: ATT) and their confidence intervals relative to the average outcome (incidence rate per capita) in the control group (Figure 1).” This sentence seems incomplete. Perhaps delete the part after “relative to...“.
   > I have added a semicolon and a comma to clarify the structure of the sentence. This sentence is meant to indicate that both the ATC (or ATT) and their confidence intervals were rescaled to a relative value, where the incidence rate per capita in the control group is the reference.

 

1. Page 3: Spell out ESS and provide a bit more context of what it is.

***> I have spelled it out with a brief explanation and citation: “The effective sample size (ESS; a proxy measure for the amount of information contained in weighted samples (Shook-Sa and Hudgens))…”***
